# (*Z*)-*N*-*tert*-Butyl-2-(4-meth­oxy­anilino)-*N*′-(4-meth­oxy­phen­yl)-2-phenyl­acetimidamide

**DOI:** 10.1107/S1600536813012877

**Published:** 2013-05-18

**Authors:** Sue A. Roberts, Biswajit Saha, Brendan Frett, Hong-Yu Li

**Affiliations:** a Department of Chemistry and Biochemistry, 1306 E University Boulevard, The University of Arizona, Tucson, AZ 85721, USA; b College of Pharmacy, Department of Pharmacology and Toxicology, The University of Arizona, Tucson, AZ 85721, USA; c Bio5 Oro Valley, The University of Arizona, Oro Valley, AZ 85737, USA

## Abstract

In the crystal of the title compound, C_26_H_31_N_3_O_2_, pairs of N—H⋯O hydrogen bonds link molecules, forming inversion dimers, which enclose an *R*_2_^2^(20) ring motif. One N atom does not form hydrogen bonds and lies in a hydro­phobic pocket with closest inter­molecular contacts of 4.196 (2) and 4.262 (2) Å.

## Related literature

For the synthesis of the title compound, and a discussion of the use of the three-component Ugi reaction to synthesize amidines, see: Saha *et al.* (2013 ▶).
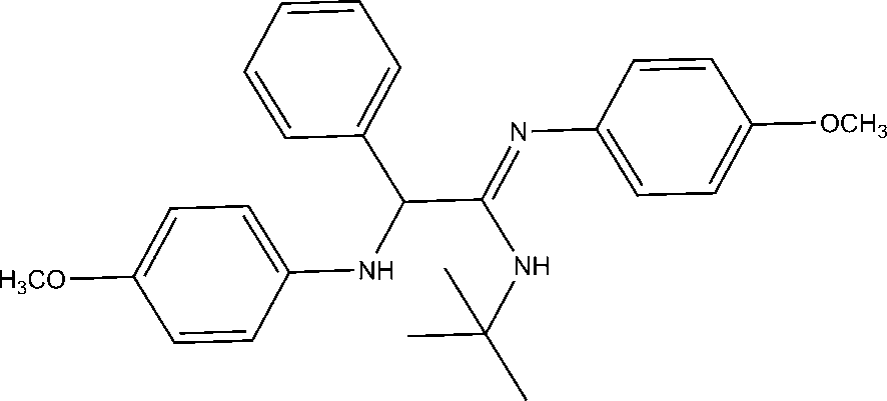


## Experimental

### 

#### Crystal data

C_26_H_31_N_3_O_2_
*M* *_r_* = 417.54
Triclinic, 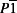

*a* = 10.0804 (17) Å
*b* = 10.5784 (17) Å
*c* = 11.1573 (18) Å
α = 80.982 (4)°
β = 85.152 (4)°
γ = 80.270 (4)°
*V* = 1156.1 (3) Å^3^
*Z* = 2
Mo *K*α radiation
μ = 0.08 mm^−1^
*T* = 100 K
0.20 × 0.20 × 0.10 mm

#### Data collection

Bruker Kappa APEXII DUO CCD diffractometer
Absorption correction: multi-scan (*SADABS*; Bruker, 2009 ▶) *T*_min_ = 0.89, *T*_max_ = 0.99
32612 measured reflections
6607 independent reflections
5476 reflections with *I* > 2σ(*I*)
*R*_int_ = 0.019

#### Refinement

*R*[*F* ^2^ > 2σ(*F* ^2^)] = 0.043
*wR*(*F* ^2^) = 0.106
*S* = 0.94
6607 reflections
289 parameters
H atoms treated by a mixture of independent and constrained refinement
Δρ_max_ = 0.36 e Å^−3^
Δρ_min_ = −0.38 e Å^−3^

### 

Data collection: *APEX2* (Bruker, 2009 ▶); cell refinement: *SAINT* (Bruker, 2009 ▶); data reduction: *SAINT*; program(s) used to solve structure: *SHELXS97* (Sheldrick, 2008 ▶); program(s) used to refine structure: *SHELXL97* (Sheldrick, 2008 ▶) and *OLEX2* (Dolomanov *et al.*, 2009 ▶); molecular graphics: *ORTEP-3 for Windows* (Farrugia, 2012 ▶) and *Mercury* (Macrae *et al.*, 2006 ▶); software used to prepare material for publication: *publCIF* (Westrip, 2010 ▶).

## Supplementary Material

Crystal structure: contains datablock(s) global, I. DOI: 10.1107/S1600536813012877/pk2477sup1.cif

Structure factors: contains datablock(s) I. DOI: 10.1107/S1600536813012877/pk2477Isup2.hkl

Supplementary material file. DOI: 10.1107/S1600536813012877/pk2477Isup3.cml

Additional supplementary materials:  crystallographic information; 3D view; checkCIF report

## Figures and Tables

**Table 1:** Hydrogen-bond geometry (Å, °)

*D*—H⋯*A*	*D*—H	H⋯*A*	*D*⋯*A*	*D*—H⋯*A*
N2—H2⋯O1^i^	0.887 (15)	2.328 (15)	3.1698 (13)	158.5 (12)

Symmetry code: (i) 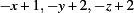.

**Fig. 1.**
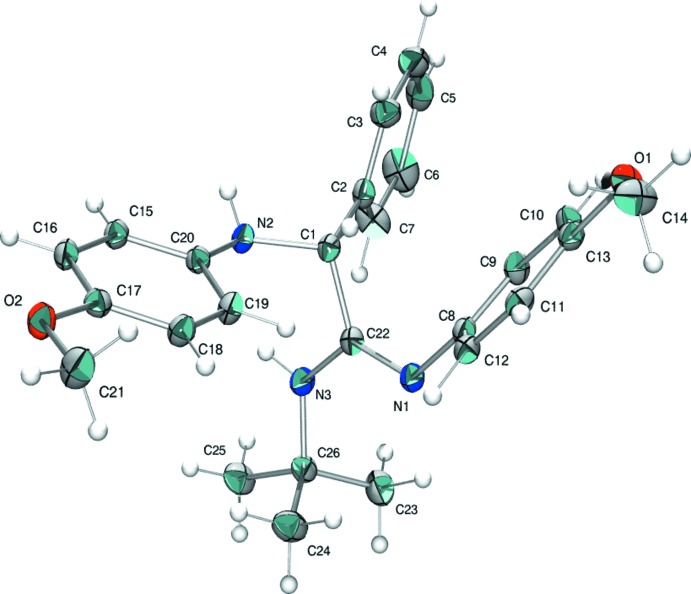
The molecular structure of the title compound. Non-hydrogen atoms are shown as 50% probability ellipsoids.

**Fig. 2.**
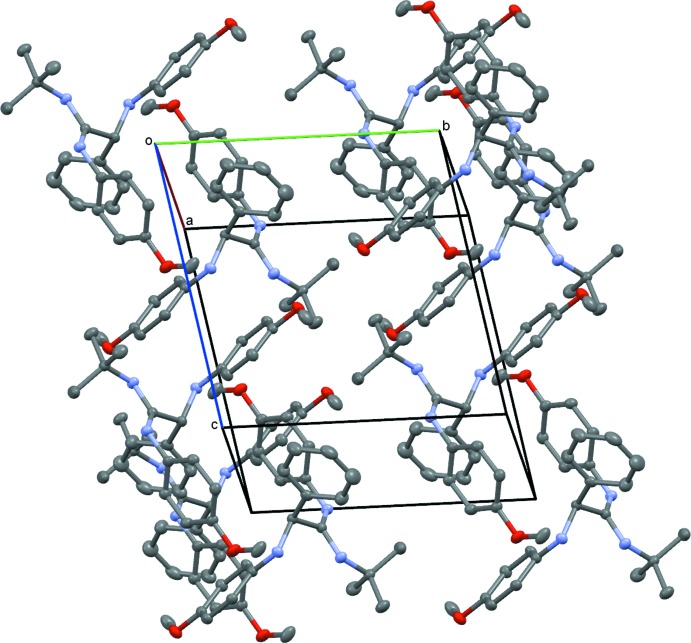
View showing unit cell packing. Unit cell axes are labeled in the figure.

## supplementary crystallographic information

### Comment

The title compound was prepared as a secondary product of the three component Ugi reaction (Saha *et al.*, 2013). The central C1—C22 single bond has four bulky substituents arranged, roughly, as if pointing at the corners of a tetrahedron. The angle between the planes containing the substituents, defined by N1—C22—N3 and C2—C1—C20, is 72.41 (1)°. Pairs of hydrogen bonds between N2 and O1 [3.170 (1) Å] connect two molecules across an inversion center, creating a cavity around the inversion center with a diameter of about 5 Å. The closest contact across the inversion center involving atoms not involved in the hydrogen bonding is 3.151 (3) Å between the hydrogen bonded to C3 and its symmetry equivalent (symmetry transformation -*x* + 1, -*y* + 2, -*z* + 2). The hydrogen bonding graph set is R^2^_2_(20). Nitrogen N3 is unusual in that, although protonated, it does not form hydrogen bonds with acceptor atoms. Instead, it lies in a hydrophobic cavity with closest intermolecular contacts of 4.196 (2) Å and 4.262 (2) Å to C15 and C16 respectively.

### Experimental

The compound was synthesized as previously reported [compound 5a in (Saha *et al.*, 2013)]. The crude residue was purified by silica gel column chromatography using (10–30%) ethylacetate-hexane to obtain the pure product. The pure compound was dissolved in 50% ethylacetate-hexane and kept at room temperature for 2 days during which crystals formed.

### Refinement

All hydrogen atoms were visible in a difference Fourier map and, except for H2 were added at calculated positions. Bond distances are set to 0.95 Å for aromatic carbon-hydrogen bonds, 0.98 Å for methyl group carbon-hydrogen bonds and 0.88 Å for nitrogen-hydrogen bonds. Thermal parameters for hydrogen atoms were set to 1.2 times the isotropic equivalent thermal parameter of the atom to which the hydrogen atom is bonded, except for methyl group hydrogen atoms where the thermal parameter was set to 1.5 times the isotropic equivalent thermal parameter of the carbon atom the hydrogen atom is bonded to. The positional and isotropic thermal parameters of H2 were refined.

### Crystal data

**Table:**

C_26_H_31_N_3_O_2_	*Z* = 2
*M**_r_* = 417.54	*F*(000) = 448
Triclinic, *P*1	*D*_x_ = 1.199 Mg m^−^^3^
*a* = 10.0804 (17) Å	Mo *K*α radiation, λ = 0.71073 Å
*b* = 10.5784 (17) Å	Cell parameters from 9952 reflections
*c* = 11.1573 (18) Å	θ = 2.9–29.8°
α = 80.982 (4)°	µ = 0.08 mm^−^^1^
β = 85.152 (4)°	*T* = 100 K
γ = 80.270 (4)°	Block, colourless
*V* = 1156.1 (3) Å^3^	0.20 × 0.20 × 0.10 mm

### Data collection

**Table:**

Bruker Kappa APEXII DUO CCD diffractometer	6607 independent reflections
Radiation source: fine-focus sealed tube	5476 reflections with *I* > 2σ(*I*)
Graphite monochromator	*R*_int_ = 0.019
Detector resolution: 8.3333 pixels mm^-1^	θ_max_ = 29.9°, θ_min_ = 1.9°
φ and ω scans	*h* = −14→11
Absorption correction: multi-scan (*SADABS*; Bruker, 2009)	*k* = −14→12
*T*_min_ = 0.89, *T*_max_ = 0.99	*l* = −15→13
32612 measured reflections	

### Refinement

**Table:**

Refinement on *F*^2^	Primary atom site location: structure-invariant direct methods
Least-squares matrix: full	Secondary atom site location: difference Fourier map
*R*[*F*^2^ > 2σ(*F*^2^)] = 0.043	Hydrogen site location: inferred from neighbouring sites
*wR*(*F*^2^) = 0.106	H atoms treated by a mixture of independent and constrained refinement
*S* = 0.94	*w* = 1/[σ^2^(*F*_o_^2^) + (0.0397*P*)^2^ + 0.6486*P*] where *P* = (*F*_o_^2^ + 2*F*_c_^2^)/3
6607 reflections	(Δ/σ)_max_ = 0.001
289 parameters	Δρ_max_ = 0.36 e Å^−^^3^
0 restraints	Δρ_min_ = −0.38 e Å^−^^3^

### Special details

**Table:**

Geometry. All e.s.d.'s (except the e.s.d. in the dihedral angle between two l.s. planes) are estimated using the full covariance matrix. The cell e.s.d.'s are taken into account individually in the estimation of e.s.d.'s in distances, angles and torsion angles; correlations between e.s.d.'s in cell parameters are only used when they are defined by crystal symmetry. An approximate (isotropic) treatment of cell e.s.d.'s is used for estimating e.s.d.'s involving l.s. planes.
Refinement. Refinement of *F*^2^ against ALL reflections. The weighted *R*-factor *wR* and goodness of fit *S* are based on *F*^2^, conventional *R*-factors *R* are based on *F*, with *F* set to zero for negative *F*^2^. The threshold expression of *F*^2^ > σ(*F*^2^) is used only for calculating *R*-factors(gt) *etc*. and is not relevant to the choice of reflections for refinement. *R*-factors based on *F*^2^ are statistically about twice as large as those based on *F*, and *R*- factors based on ALL data will be even larger.

### Fractional atomic coordinates and isotropic or equivalent isotropic displacement parameters (Å^2^)

**Table:**

	*x*	*y*	*z*	*U*_iso_*/*U*_eq_	
C20	0.37947 (10)	1.03405 (9)	0.68022 (9)	0.01884 (19)	
C11	0.15660 (10)	0.92039 (10)	1.16518 (9)	0.0213 (2)	
H11	0.0822	0.9836	1.1842	0.026*	
C18	0.15655 (11)	1.15884 (10)	0.70473 (10)	0.0226 (2)	
H18	0.0708	1.1695	0.7475	0.027*	
C15	0.40664 (11)	1.12635 (10)	0.57996 (9)	0.02047 (19)	
H15	0.4919	1.1155	0.5363	0.025*	
C16	0.31125 (11)	1.23257 (10)	0.54406 (9)	0.0219 (2)	
H16	0.3313	1.2940	0.4761	0.026*	
C22	0.35777 (10)	0.75795 (9)	0.82814 (9)	0.01868 (19)	
C10	0.36795 (11)	0.78673 (11)	1.21611 (9)	0.0228 (2)	
H10	0.4377	0.7582	1.2709	0.027*	
C12	0.16277 (10)	0.86653 (10)	1.05826 (9)	0.0212 (2)	
H12	0.0906	0.8921	1.0057	0.025*	
C19	0.25281 (11)	1.05135 (10)	0.74063 (10)	0.0224 (2)	
H19	0.2315	0.9890	0.8074	0.027*	
C8	0.27219 (10)	0.77613 (10)	1.02648 (9)	0.01957 (19)	
C1	0.46163 (10)	0.84821 (9)	0.83240 (9)	0.01839 (18)	
H1	0.4260	0.9082	0.8927	0.022*	
C17	0.18548 (10)	1.25005 (10)	0.60709 (9)	0.0206 (2)	
C9	0.37371 (11)	0.73460 (10)	1.10887 (9)	0.0223 (2)	
H9	0.4473	0.6700	1.0910	0.027*	
C2	0.59808 (10)	0.77504 (10)	0.87123 (9)	0.0209 (2)	
C26	0.27437 (11)	0.63246 (10)	0.68176 (9)	0.0220 (2)	
C3	0.67295 (12)	0.83256 (11)	0.93977 (11)	0.0280 (2)	
H3	0.6367	0.9144	0.9636	0.034*	
C24	0.12887 (12)	0.70234 (13)	0.67994 (12)	0.0318 (3)	
H24A	0.0962	0.7183	0.7623	0.048*	
H24B	0.0722	0.6484	0.6505	0.048*	
H24C	0.1249	0.7852	0.6258	0.048*	
C7	0.65156 (13)	0.65363 (12)	0.83952 (12)	0.0329 (3)	
H7	0.6010	0.6121	0.7938	0.039*	
C25	0.32629 (13)	0.61040 (12)	0.55297 (10)	0.0299 (2)	
H25A	0.3167	0.6936	0.4991	0.045*	
H25B	0.2739	0.5524	0.5239	0.045*	
H25C	0.4215	0.5710	0.5532	0.045*	
C4	0.80027 (13)	0.77209 (13)	0.97403 (12)	0.0336 (3)	
H4	0.8505	0.8127	1.0209	0.040*	
C21	−0.02644 (12)	1.38154 (12)	0.63778 (12)	0.0333 (3)	
H21A	−0.0784	1.3119	0.6344	0.050*	
H21B	−0.0785	1.4649	0.6052	0.050*	
H21C	−0.0078	1.3829	0.7223	0.050*	
C14	0.15403 (12)	1.02124 (13)	1.38420 (12)	0.0335 (3)	
H14A	0.1460	1.0989	1.3229	0.050*	
H14B	0.1674	1.0449	1.4633	0.050*	
H14C	0.0715	0.9827	1.3897	0.050*	
C5	0.85386 (12)	0.65322 (13)	0.94015 (12)	0.0350 (3)	
H5	0.9419	0.6128	0.9616	0.042*	
C23	0.28606 (16)	0.50314 (12)	0.76560 (12)	0.0366 (3)	
H23A	0.3800	0.4597	0.7629	0.055*	
H23B	0.2285	0.4483	0.7388	0.055*	
H23C	0.2573	0.5183	0.8490	0.055*	
C6	0.77862 (14)	0.59305 (14)	0.87460 (13)	0.0405 (3)	
H6	0.8141	0.5098	0.8535	0.049*	
O2	0.09729 (8)	1.35968 (7)	0.56724 (7)	0.02582 (17)	
O1	0.26631 (8)	0.92977 (8)	1.35034 (7)	0.02582 (17)	
N2	0.47953 (9)	0.92663 (8)	0.71356 (8)	0.01953 (17)	
N3	0.36308 (9)	0.71424 (9)	0.71966 (8)	0.02124 (18)	
H3A	0.4268	0.7374	0.6658	0.025*	
N1	0.27336 (9)	0.72595 (9)	0.91628 (8)	0.02123 (18)	
C13	0.26038 (10)	0.88065 (10)	1.24380 (9)	0.0204 (2)	
H2	0.5625 (15)	0.9449 (14)	0.6977 (13)	0.027 (3)*	

### Atomic displacement parameters (Å^2^)

**Table:**

	*U*^11^	*U*^22^	*U*^33^	*U*^12^	*U*^13^	*U*^23^
C20	0.0210 (4)	0.0187 (4)	0.0171 (4)	−0.0043 (4)	−0.0002 (3)	−0.0025 (3)
C11	0.0189 (4)	0.0221 (5)	0.0226 (5)	−0.0038 (4)	0.0033 (4)	−0.0041 (4)
C18	0.0203 (5)	0.0226 (5)	0.0236 (5)	−0.0037 (4)	0.0019 (4)	−0.0008 (4)
C15	0.0230 (5)	0.0221 (5)	0.0164 (4)	−0.0057 (4)	0.0020 (4)	−0.0025 (3)
C16	0.0275 (5)	0.0212 (5)	0.0168 (4)	−0.0066 (4)	0.0000 (4)	0.0002 (3)
C22	0.0206 (4)	0.0177 (4)	0.0170 (4)	−0.0019 (3)	−0.0004 (3)	−0.0022 (3)
C10	0.0227 (5)	0.0274 (5)	0.0177 (4)	−0.0019 (4)	−0.0007 (4)	−0.0033 (4)
C12	0.0198 (4)	0.0246 (5)	0.0191 (4)	−0.0052 (4)	0.0003 (4)	−0.0017 (4)
C19	0.0229 (5)	0.0210 (5)	0.0212 (5)	−0.0034 (4)	0.0025 (4)	0.0016 (4)
C8	0.0219 (5)	0.0212 (5)	0.0160 (4)	−0.0072 (4)	0.0026 (4)	−0.0018 (3)
C1	0.0202 (4)	0.0185 (4)	0.0160 (4)	−0.0032 (3)	0.0014 (3)	−0.0021 (3)
C17	0.0228 (5)	0.0189 (5)	0.0205 (4)	−0.0036 (4)	−0.0036 (4)	−0.0022 (4)
C9	0.0231 (5)	0.0236 (5)	0.0186 (4)	−0.0005 (4)	0.0014 (4)	−0.0035 (4)
C2	0.0204 (4)	0.0227 (5)	0.0176 (4)	−0.0023 (4)	0.0017 (4)	0.0003 (4)
C26	0.0253 (5)	0.0227 (5)	0.0190 (4)	−0.0051 (4)	−0.0019 (4)	−0.0050 (4)
C3	0.0265 (5)	0.0246 (5)	0.0331 (6)	−0.0050 (4)	−0.0047 (4)	−0.0024 (4)
C24	0.0237 (5)	0.0407 (7)	0.0322 (6)	−0.0036 (5)	−0.0011 (4)	−0.0112 (5)
C7	0.0319 (6)	0.0317 (6)	0.0344 (6)	0.0050 (5)	−0.0052 (5)	−0.0120 (5)
C25	0.0323 (6)	0.0362 (6)	0.0235 (5)	−0.0045 (5)	−0.0005 (4)	−0.0132 (4)
C4	0.0283 (6)	0.0347 (6)	0.0371 (6)	−0.0080 (5)	−0.0088 (5)	0.0036 (5)
C21	0.0233 (5)	0.0299 (6)	0.0406 (7)	0.0017 (4)	−0.0003 (5)	0.0062 (5)
C14	0.0255 (5)	0.0433 (7)	0.0359 (6)	−0.0052 (5)	0.0066 (5)	−0.0234 (5)
C5	0.0249 (5)	0.0396 (7)	0.0336 (6)	0.0028 (5)	−0.0021 (5)	0.0080 (5)
C23	0.0594 (8)	0.0243 (6)	0.0291 (6)	−0.0136 (6)	−0.0126 (6)	−0.0005 (4)
C6	0.0363 (7)	0.0366 (7)	0.0433 (7)	0.0128 (5)	−0.0028 (6)	−0.0091 (6)
O2	0.0252 (4)	0.0223 (4)	0.0267 (4)	−0.0005 (3)	−0.0026 (3)	0.0034 (3)
O1	0.0240 (4)	0.0337 (4)	0.0215 (4)	−0.0035 (3)	0.0026 (3)	−0.0124 (3)
N2	0.0194 (4)	0.0206 (4)	0.0172 (4)	−0.0035 (3)	0.0028 (3)	−0.0002 (3)
N3	0.0237 (4)	0.0240 (4)	0.0171 (4)	−0.0065 (3)	0.0030 (3)	−0.0056 (3)
N1	0.0246 (4)	0.0233 (4)	0.0164 (4)	−0.0062 (3)	0.0010 (3)	−0.0033 (3)
C13	0.0213 (5)	0.0235 (5)	0.0173 (4)	−0.0069 (4)	0.0035 (4)	−0.0047 (4)

### Geometric parameters (Å, º)

**Table:**

C20—C15	1.4057 (14)	C26—C25	1.5264 (15)
C20—C19	1.3901 (14)	C26—C23	1.5243 (16)
C20—N2	1.4108 (13)	C26—N3	1.4721 (13)
C11—H11	0.9500	C3—H3	0.9500
C11—C12	1.3930 (14)	C3—C4	1.3897 (17)
C11—C13	1.3897 (15)	C24—H24A	0.9800
C18—H18	0.9500	C24—H24B	0.9800
C18—C19	1.3946 (15)	C24—H24C	0.9800
C18—C17	1.3836 (14)	C7—H7	0.9500
C15—H15	0.9500	C7—C6	1.3903 (18)
C15—C16	1.3807 (15)	C25—H25A	0.9800
C16—H16	0.9500	C25—H25B	0.9800
C16—C17	1.3953 (15)	C25—H25C	0.9800
C22—C1	1.5397 (14)	C4—H4	0.9500
C22—N3	1.3556 (12)	C4—C5	1.3787 (19)
C22—N1	1.2873 (13)	C21—H21A	0.9800
C10—H10	0.9500	C21—H21B	0.9800
C10—C9	1.3869 (14)	C21—H21C	0.9800
C10—C13	1.3906 (15)	C21—O2	1.4220 (15)
C12—H12	0.9500	C14—H14A	0.9800
C12—C8	1.3930 (15)	C14—H14B	0.9800
C19—H19	0.9500	C14—H14C	0.9800
C8—C9	1.4021 (14)	C14—O1	1.4256 (14)
C8—N1	1.4120 (13)	C5—H5	0.9500
C1—H1	1.0000	C5—C6	1.385 (2)
C1—C2	1.5213 (14)	C23—H23A	0.9800
C1—N2	1.4643 (13)	C23—H23B	0.9800
C17—O2	1.3747 (12)	C23—H23C	0.9800
C9—H9	0.9500	C6—H6	0.9500
C2—C3	1.3868 (15)	O1—C13	1.3794 (12)
C2—C7	1.3934 (16)	N2—H2	0.887 (15)
C26—C24	1.5270 (16)	N3—H3A	0.8800
			
C15—C20—N2	119.16 (9)	C26—C24—H24A	109.5
C19—C20—C15	118.08 (9)	C26—C24—H24B	109.5
C19—C20—N2	122.74 (9)	C26—C24—H24C	109.5
C12—C11—H11	120.3	H24A—C24—H24B	109.5
C13—C11—H11	120.3	H24A—C24—H24C	109.5
C13—C11—C12	119.31 (9)	H24B—C24—H24C	109.5
C19—C18—H18	119.9	C2—C7—H7	119.9
C17—C18—H18	119.9	C6—C7—C2	120.22 (12)
C17—C18—C19	120.28 (10)	C6—C7—H7	119.9
C20—C15—H15	119.5	C26—C25—H25A	109.5
C16—C15—C20	120.96 (10)	C26—C25—H25B	109.5
C16—C15—H15	119.5	C26—C25—H25C	109.5
C15—C16—H16	119.8	H25A—C25—H25B	109.5
C15—C16—C17	120.31 (9)	H25A—C25—H25C	109.5
C17—C16—H16	119.8	H25B—C25—H25C	109.5
N3—C22—C1	112.76 (8)	C3—C4—H4	119.9
N1—C22—C1	124.98 (9)	C5—C4—C3	120.11 (12)
N1—C22—N3	122.26 (9)	C5—C4—H4	119.9
C9—C10—H10	119.9	H21A—C21—H21B	109.5
C9—C10—C13	120.25 (10)	H21A—C21—H21C	109.5
C13—C10—H10	119.9	H21B—C21—H21C	109.5
C11—C12—H12	119.2	O2—C21—H21A	109.5
C11—C12—C8	121.63 (10)	O2—C21—H21B	109.5
C8—C12—H12	119.2	O2—C21—H21C	109.5
C20—C19—C18	120.99 (9)	H14A—C14—H14B	109.5
C20—C19—H19	119.5	H14A—C14—H14C	109.5
C18—C19—H19	119.5	H14B—C14—H14C	109.5
C12—C8—C9	118.01 (9)	O1—C14—H14A	109.5
C12—C8—N1	119.03 (9)	O1—C14—H14B	109.5
C9—C8—N1	122.89 (9)	O1—C14—H14C	109.5
C22—C1—H1	108.1	C4—C5—H5	120.2
C2—C1—C22	112.85 (8)	C4—C5—C6	119.59 (11)
C2—C1—H1	108.1	C6—C5—H5	120.2
N2—C1—C22	110.37 (8)	C26—C23—H23A	109.5
N2—C1—H1	108.1	C26—C23—H23B	109.5
N2—C1—C2	109.12 (8)	C26—C23—H23C	109.5
C18—C17—C16	119.35 (9)	H23A—C23—H23B	109.5
O2—C17—C18	123.94 (10)	H23A—C23—H23C	109.5
O2—C17—C16	116.71 (9)	H23B—C23—H23C	109.5
C10—C9—C8	120.74 (10)	C7—C6—H6	119.8
C10—C9—H9	119.6	C5—C6—C7	120.43 (12)
C8—C9—H9	119.6	C5—C6—H6	119.8
C3—C2—C1	118.49 (9)	C17—O2—C21	116.18 (8)
C3—C2—C7	118.73 (10)	C13—O1—C14	117.04 (9)
C7—C2—C1	122.78 (10)	C20—N2—C1	118.01 (8)
C25—C26—C24	109.45 (9)	C20—N2—H2	112.8 (9)
C23—C26—C24	111.49 (10)	C1—N2—H2	113.0 (9)
C23—C26—C25	109.80 (10)	C22—N3—C26	127.34 (9)
N3—C26—C24	110.61 (9)	C22—N3—H3A	116.3
N3—C26—C25	105.50 (9)	C26—N3—H3A	116.3
N3—C26—C23	109.82 (9)	C22—N1—C8	119.35 (9)
C2—C3—H3	119.6	C11—C13—C10	119.96 (9)
C2—C3—C4	120.89 (11)	O1—C13—C11	124.18 (9)
C4—C3—H3	119.6	O1—C13—C10	115.85 (9)
			
C20—C15—C16—C17	−0.05 (16)	C9—C8—N1—C22	−72.76 (14)
C11—C12—C8—C9	3.56 (15)	C2—C1—N2—C20	−157.97 (9)
C11—C12—C8—N1	−179.35 (9)	C2—C3—C4—C5	−0.11 (19)
C18—C17—O2—C21	4.27 (15)	C2—C7—C6—C5	−0.7 (2)
C15—C20—C19—C18	−1.56 (16)	C3—C2—C7—C6	−1.07 (19)
C15—C20—N2—C1	166.59 (9)	C3—C4—C5—C6	−1.7 (2)
C15—C16—C17—C18	−1.01 (16)	C24—C26—N3—C22	62.76 (14)
C15—C16—C17—O2	178.62 (9)	C7—C2—C3—C4	1.49 (17)
C16—C17—O2—C21	−175.34 (10)	C25—C26—N3—C22	−178.98 (10)
C22—C1—C2—C3	−145.84 (10)	C4—C5—C6—C7	2.1 (2)
C22—C1—C2—C7	35.00 (14)	C14—O1—C13—C11	3.97 (15)
C22—C1—N2—C20	77.49 (11)	C14—O1—C13—C10	−176.82 (10)
C12—C11—C13—C10	−0.89 (15)	C23—C26—N3—C22	−60.72 (14)
C12—C11—C13—O1	178.29 (9)	N2—C20—C15—C16	179.73 (9)
C12—C8—C9—C10	−2.92 (15)	N2—C20—C19—C18	−179.91 (10)
C12—C8—N1—C22	110.29 (12)	N2—C1—C2—C3	91.07 (11)
C19—C20—C15—C16	1.32 (15)	N2—C1—C2—C7	−88.08 (12)
C19—C20—N2—C1	−15.07 (14)	N3—C22—C1—C2	−87.41 (10)
C19—C18—C17—C16	0.77 (16)	N3—C22—C1—N2	34.97 (11)
C19—C18—C17—O2	−178.83 (10)	N3—C22—N1—C8	−179.30 (9)
C1—C22—N3—C26	−176.49 (9)	N1—C22—C1—C2	92.83 (12)
C1—C22—N1—C8	0.44 (15)	N1—C22—C1—N2	−144.79 (10)
C1—C2—C3—C4	−177.70 (10)	N1—C22—N3—C26	3.28 (16)
C1—C2—C7—C6	178.08 (11)	N1—C8—C9—C10	−179.89 (10)
C17—C18—C19—C20	0.53 (16)	C13—C11—C12—C8	−1.69 (15)
C9—C10—C13—C11	1.50 (16)	C13—C10—C9—C8	0.45 (16)
C9—C10—C13—O1	−177.75 (9)		

### Hydrogen-bond geometry (Å, º)

**Table:**

*D*—H···*A*	*D*—H	H···*A*	*D*···*A*	*D*—H···*A*
N2—H2···O1^i^	0.887 (15)	2.328 (15)	3.1698 (13)	158.5 (12)

Symmetry code: (i) −*x*+1, −*y*+2, −*z*+2.
